# Blockchain in Smart Grids: A Review on Different Use Cases

**DOI:** 10.3390/s19224862

**Published:** 2019-11-08

**Authors:** Tejasvi Alladi, Vinay Chamola, Joel J. P. C. Rodrigues, Sergei A. Kozlov

**Affiliations:** 1 Birla Institute of Technology and Science, Pilani 333031, India; p20170433@pilani.bits-pilani.ac.in (T.A.); vinay.chamola@pilani.bits-pilani.ac.in (V.C.); 2 Federal University of Piauí, Teresina-PI 64049-550, Brazil; 3 Instituto de Telecomunicações, 1049-001 Lisboa, Portugal; 4 International Institute of Photonics and Optoinformatics, ITMO University, St. Petersburg 197101, Russia; kozlov@mail.ifmo.ru

**Keywords:** blockchain, smart grid, Internet of Things, security and privacy, Peer-to-Peer trading, Wireless Sensor Networks

## Abstract

With the integration of Wireless Sensor Networks and the Internet of Things, the smart grid is being projected as a solution for the challenges regarding electricity supply in the future. However, security and privacy issues in the consumption and trading of electricity data pose serious challenges in the adoption of the smart grid. To address these challenges, blockchain technology is being researched for applicability in the smart grid. In this paper, important application areas of blockchain in the smart grid are discussed. One use case of each area is discussed in detail, suggesting a suitable blockchain architecture, a sample block structure and the potential blockchain technicalities employed in it. The blockchain can be used for peer-to-peer energy trading, where a credit-based payment scheme can enhance the energy trading process. Efficient data aggregation schemes based on the blockchain technology can be used to overcome the challenges related to privacy and security in the grid. Energy distribution systems can also use blockchain to remotely control energy flow to a particular area by monitoring the usage statistics of that area. Further, blockchain-based frameworks can also help in the diagnosis and maintenance of smart grid equipment. We also discuss several commercial implementations of blockchain in the smart grid. Finally, various challenges to be addressed for integrating these two technologies are discussed.

## 1. Introduction

Smart grids are currently advancing technologically at a very fast pace by leveraging the benefits offered by Wireless Sensor Networks (WSNs) and the Internet of Things (IoT). They offer optimization in energy production and consumption by the adoption of intelligent systems that can monitor and communicate with each other [1,2,3]. Automation of the smart sensor-based metering system by using Advanced Metering Devices (AMI) leads to a lesser requirement of manpower and more accuracy. Thus, by making the grid more intelligent, efficient energy utilization is achieved [4,5]. Smart grids also promise more efficient tapping of renewable sources of energy by offering technological support for the transfer of energy between local energy producers and consumers. The consumers who can harvest renewable sources of energy such as sunlight using rooftop solar panels can become producers-cum-consumers (prosumers) by selling their surplus energy either to neighboring consumers or to the grid. This promotes consumers to utilize renewable sources of energy [6]. Since the energy demand is ever-growing and there are multiple sources of energy, the need for a decentralized energy management system has arisen [7]. The system should be able to manage the individual transactions between the users as well as between the user and the grid without any tampering of data or loss of information. Integrating distributed renewable energy resources whose power generation is highly fluctuating makes it very challenging for the utilities to estimate the state of the system. Some works [8,9,10] have proposed novel Kalman filter-based approaches for accurate microgrid state estimation and control for the smart grids. Their models encourage consumers to use environment-friendly renewable energy sources which will lead to many benefits such as line-loss reduction, reliability, energy efficiency, etc. The authors of [11] discussed energy demand reduction of the utilities and consumers and smart energy management while considering the ever-growing renewable energy integration. Another issue that hinders an efficient grid management system is the requirement of third parties for the supply and distribution of energy. Third-party involvement always increases the cost of operation drastically and paves the way for erroneous transactions, intentionally or otherwise. This is where blockchain offers a promising solution to these existing issues of the smart grid [12,13].

The adoption of blockchain technology allows the grid network to decentralize its operations. That means the decision making and the transaction flows do not need to be channeled through a centralized system that is inclusive of third parties, e.g., mediators, banks, etc. The record of transactions is stored in all or selected nodes involved in the operation of the network depending on the type of blockchain used [14,15]. The transactions of buying and selling of energy across users no longer needs to go through the procedures of a bank but rather can be done through a computer program by validating the required pre-determined clauses of the transaction [16]. Blockchain technology among various other benefits helps in setting up real-time energy markets and identity preserving transactions at much lower costs due to a simplified trading framework [17,18]. The computation and power consumption of IoT devices are important challenges restricting the application of blockchain in IoT and smart grid. The authors of [19] proposed a decentralized on-demand energy supply architecture for miners in the IoT network, using microgrids to provide renewable energy for mining in the IoT devices. This paper identifies some of the various scenarios in which blockchain can be incorporated in the smart grid, and discusses the various technological aspects about each scenario.

The main contributions of this paper are:

- We discuss major applications of blockchain in smart grids, giving details such as blockchain architecture, sample block structure and blockchain-related technologies employed in each application area.
- A table summarizing these application areas with important technical details is also presented after a discussion of the application areas.
- We then discuss commercial implementations of blockchain in the smart grid.
- We also discuss existing challenges for incorporating blockchain into the smart grid and present some future research directions.

The rest of the paper is organized as follows. Section 2 gives a brief overview of blockchain technology. In Section 3, important application areas of blockchain in the smart grid are discussed. Section 4 summarizes several commercial implementations of blockchain in the energy sector. In Section 5, practical challenges in the incorporation of blockchain into the smart grid are discussed. Section 6 suggests some future research directions. Finally, the paper is concluded in Section 7.

## 2. Blockchain Overview

**Figure 1:**
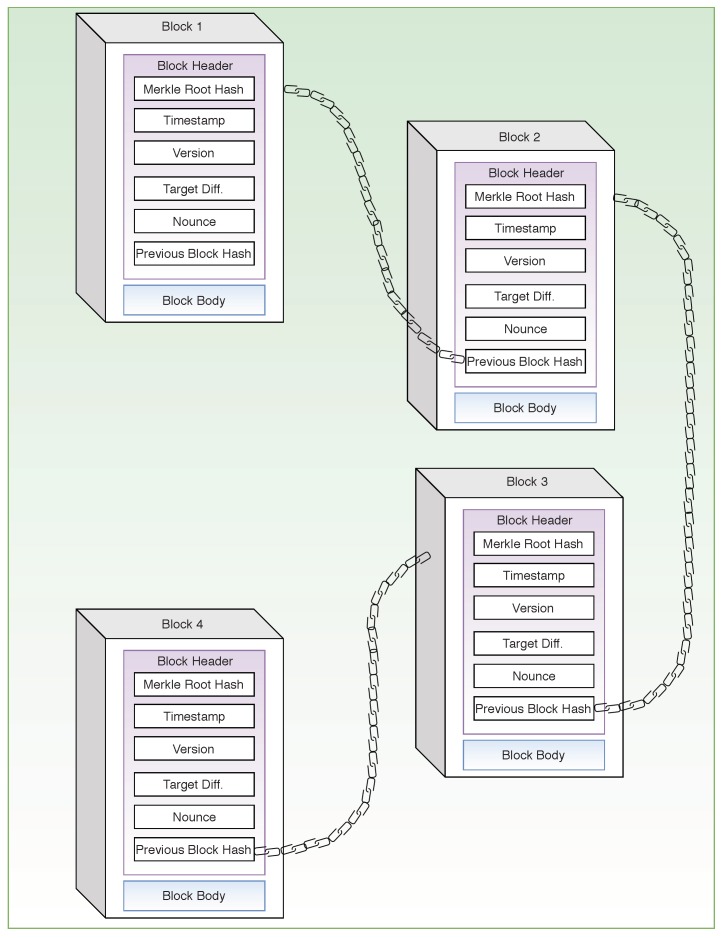
Blockchain structure.

Blockchain is a decentralized ledger meant for keeping a record of the various transactions carried out in the network right from the beginning of the chain. The ledger is shared among different nodes (also referred to as peers) that participate in the network, with each peer having its copy of the ledger. Each block in the chain is connected to the previous one using cryptographic techniques, which makes the system secure and resistive to malicious attacks and malpractices, as illustrated in Figure 1. Each node can check for the validity of the transactions and reach a consensus before adding the block to the blockchain, thus providing a high level of transparency and reliability.

### 2.1. Composition of Blockchain

Each transaction in a blockchain is verified by the participating nodes using a consensus algorithm and, if a consensus is reached upon its validity by the nodes, the transaction data are stored into structures called blocks. *Mining* is the addition of the blocks into the blockchain while the *Miners* or the *Mining Nodes* are the nodes involved in this process. A cryptographic hash function [20] links any two adjacent blocks in the blockchain, with the hash of the previous block stored in the current block. To carry out a successful attack, the attacker trying to modify a particular block in the blockchain has to ensure that all the following blocks are also modified. Since the hash of the current block is stored in the next block, modifying any field of the current block will also modify its hash. Thus, the older the target block is, more challenging it is for an attacker to modify and update the block and all the succeeding blocks until the newest block in the blockchain. Furthermore, the attacker also has to ensure that no new block has been added into the blockchain by the time his changes are reflected in the blockchain. This requires a much higher processing and hashing capability on the attacker’s end compared to the combined capability of all the miners. Therefore, such an attack on the blockchain network remains economically quite infeasible. In addition, since a copy of the complete blockchain is available with each participating node of the network, any malpractice such as modification of a block of the blockchain can be easily detected. These cryptographic security techniques thus provide data immutability to the blockchain. Each block essentially comprises of a block header and a block body. The block header contains various fields such as the previous hash, timestamp, etc. The timestamp indicates the time of the creation of a block. Version denotes the type and format of data contained in the block while the Merkle root hash is the combined hash of all the transactions that have been added into that block. Merkle trees are generated by iteratively hashing pairs of transactions until there is only one hash value left. The single hash value is called the Merkle root. Merkle root is the digital fingerprint of all the transactions stored in a particular block. Using a Merkle root, a user can securely and efficiently verify the presence of a particular transaction in a block. A nonce is an arbitrary number used by the mining nodes to change the block’s hash value to satisfy the consensus criterion of a blockchain. The block body comprising of the transaction information related to the block can be divided into two parts. The first part of the block stores information about the transactions (amount, date, time, etc.), whereas the other part stores information about the participants of the transactions. All blocks are connected to form a chain having information about the transaction history of the whole network and are shared with the whole network [21,22,23,24].

### 2.2. Classification of Blockchains

Blockchains are generally classified into three types, namely public, consortium and private blockchain. A comparison of these three types based on different parameters is summarized in Table 1.

## 3. Applications of Blockchain in Smart Grid

**Figure 2:**
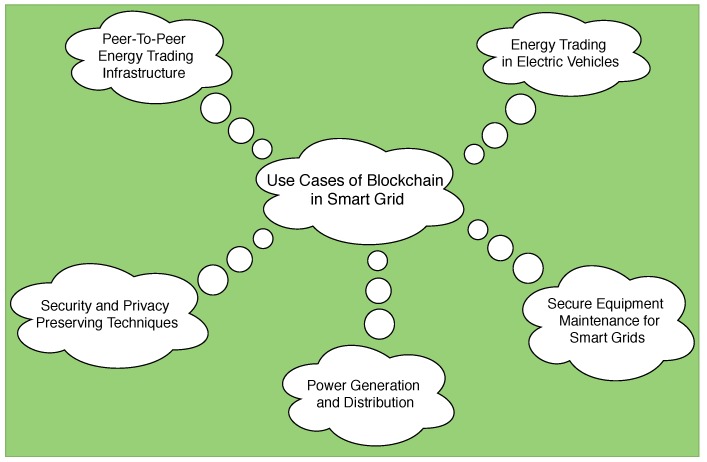
Applications of blockchain in smart grid.

Figure 2 lists important applications of blockchain in the smart grid scenario. Based on the existing surveys and reviews on blockchain applicability in IoT [32,33,34,35], in this paper, we focus on these five important application areas in smart grids where blockchain technology has been extensively researched. Each of these application areas is discussed below giving details of the blockchain architecture employed, the structure of a sample block and the different blockchain technologies used.

### 3.1. Peer-To-Peer Trading Infrastructure

A major drawback in the existing grid networks is the lack of security regarding the transactions caused by the involvement of mediators and other third parties. This hierarchical organizational trading structure of the grid leads to heavy operating costs with low efficiency of operation [36,37]. On the other hand, a blockchain-based trading infrastructure offers a decentralized platform that enables the Peer-to-Peer (P2P) trade of energy between consumers and prosumers in a secure manner. The identity privacy and security of transactions is higher in the decentralized platform compared to the traditional system. The P2P energy trade finds purpose in many applications including the Industrial Internet of Things (IIoT) and enhances the possibility of developing micro-grids leading to sustainable energy utilization [38,39]. The UK based Energy Networks Association has declared the plan to invest 17 billion Euros in the local energy markets using the smart grid [40]. Various aspects of P2P energy trade using blockchain are discussed below.

#### 3.1.1. Blockchain Architecture

**Figure 3:**
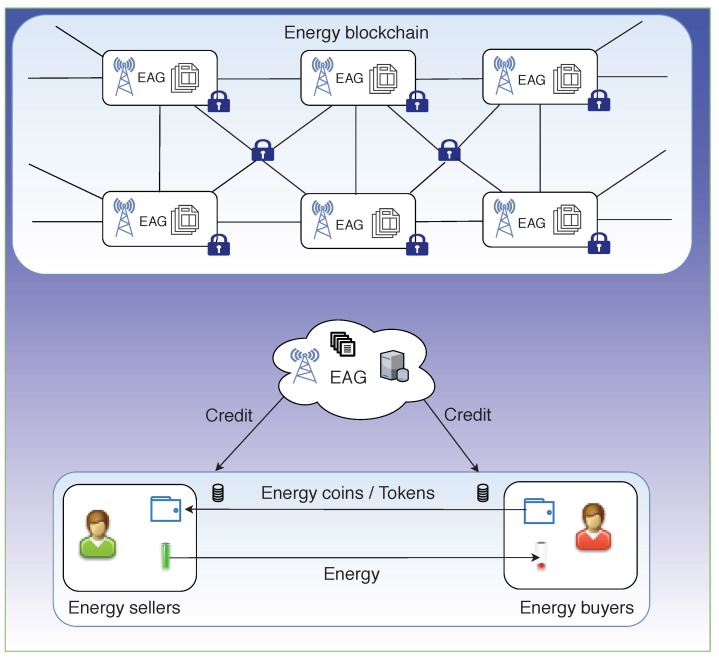
Architecture for P2P energy trading.

Based on the various state-of-the-art research works surveyed on P2P energy trading infrastructure using blockchain, the blockchain architecture for a typical P2P energy trading system can be shown as in Figure 3. This architecture is based on the reference model used in [38], in which the authors used a consortium blockchain-based secure P2P energy trading system. A comparison of several such research works is shown in Table 2. Depending on the market scenario, the required computational power and the speed of transactions, the decision regarding the choice of blockchain type to be used can vary.

A public blockchain gives a high level of transparency by providing a copy of the distributed ledger to each node, and the ability to perform consensus and validation of data. However, the disadvantage comes in the form of energy consumption and performance. A consortium blockchain, on the other hand, allows only a set of pre-authorized nodes to handle the distributed ledger or the transaction database. Only these authorized nodes are allotted high computational capabilities required to solve the consensus algorithm thereby reducing the overall power consumption and facilitating faster transactions. The authors of [38] proposed a consortium blockchain platform for facilitating a secure P2P system for energy trade in IIoT, called *energy blockchain*. The different energy nodes comprising of small scale consumers, industrial consumers, electric vehicles, etc. are given the flexibility to choose their roles as buyers/sellers or idle nodes can initiate transactions according to their requirement. A record of these transactions is stored and managed by a special authorized set of entities called *Energy Aggregators* (*EAGs*).

#### 3.1.2. Block Structure

**Figure 4:**
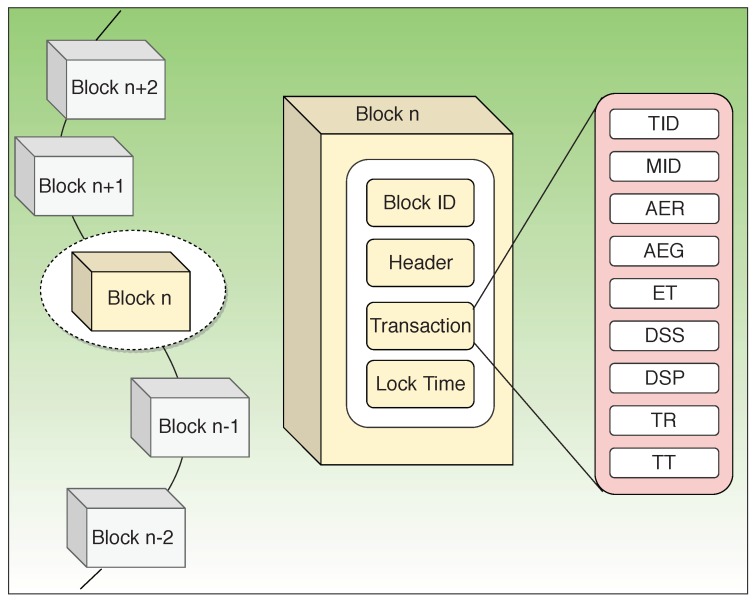
Block structure for P2P energy trading.

In the case of P2P energy transfer, a typical block in the blockchain network, as shown in Figure 4, consists of data structures that include information regarding the amount of energy used and the timestamp indicating the usage of energy usually dealing with a particular transaction [45]. The number of structures and the data included depends on the architecture adopted.

In a consortium blockchain with predefined processing and consensus nodes, the block structure consists of the Block ID for unique identification; Header, which is hashed with a Secure Hash Algorithm (SHA); Lock Time, which indicates the time of addition of that particular block into the network; and the transactions. Each transaction is generated when the buyer requests energy from the transaction servers of the supervisory nodes. The transaction part of the block structure consists of data specific to each transaction such as Transaction ID (TID), Meter ID (MID), Amount of Energy Requested (AER), Amount of Energy Granted (AEG) for the requesting buyer by the supervisory nodes based on the available energy from the sellers, Energy coins Transferred (ET) by the buyer for the transaction, Digital Signature of the Seller (DSS) indicating a successful transaction, and Digital Signature of the Processing node (DSP) indicating validation of the transaction. It also includes timestamps indicating Time of Request (TR) and Time taken for Transaction (TT).

#### 3.1.3. Technologies Used

- Virtual currency: Using blockchain, a virtual currency can be created for representing each unit of electricity. This system is highly useful in situations where renewable energy is generated at the prosumer’s end. Surplus energy available to the prosumer can be sold by engaging in transactions with other peers within the blockchain network and transferring this electrical energy into the grid. The prosumer can earn virtual currency for the energy sale at a specified price while the consumers with deficit can buy energy for their requirement with the virtual currency. The true identity of both the buyer and the seller do not need to be disclosed in such transactions using virtual coins [39,46]. Further, incentive schemes can be introduced for the promotion of renewable energy. A set of peers who contribute the most to the trade of renewable energy can be chosen by monitoring the transaction history from the blockchain ledgers and rewarded with virtual currencies.
- Credit-based transactions: Since there is some latency in the validation and addition of transactions into the blockchain, which in turn delays the release of virtual currency for the respective user, users might face a shortage of virtual currency temporarily. A credit-based transaction system helps such users in purchasing the required energy without actual possession of virtual currencies at that moment. Li et al. [38] utilized a credit-based payment scheme where each node is allotted an identity, a set of public and private keys, a certificate for unique identification, and a set of wallet addresses upon a legitimate registration onto the blockchain. Upon initialization, the wallet integrity is checked and its credit data are downloaded from the memory pool of the supervisory nodes (which store records on credit-based payments). The request from each node for the release of credit-based tokens is validated by the credit bank managed by the supervisory nodes and released if the requesting node meets the specified criteria. These tokens which are then transferred to the wallet of the node can be used to buy the required energy from other selling nodes [39,47].
- Smart contracts: These are computer codes consisting of terms of agreements under which the parties involved should interact with each other. They are finite state machines that implement some predefined instructions upon meeting a particular set of conditions or certain specified actions. Smart contracts associated with the smart meters in the grid are deployed in the blockchain. They ensure secure transactions by allowing only authentic data transfers between the smart meters and the supervisory nodes and report if any unauthorized and malicious tampering of data has occurred [47,48].

### 3.2. Energy Trading in Electric Vehicles

Electric vehicles (EVs) play an important role in the smart grid infrastructure for distributed renewable energy transportation [49,50]. There can be two sources to charge the EVs: using vehicle-to-grid (V2G) and vehicle-to-vehicle (V2V) trading. In V2V trading, EVs can trade electricity in the hotspots (charging stations or parking lots) in a P2P manner, where the discharging vehicles (with surplus electricity) discharge their energy to fulfill the electricity demand of the charging vehicles and thus balance the electricity supply–demand equilibrium. However, due to privacy concerns, discharging EVs tend to be reluctant to participate in the electricity trading market and consequently the supply and demand equilibrium among EVs becomes unbalanced. These problems with the traditional centralized electricity trading schemes which rely on intermediary parties are discussed in [51]. Hence, there is a need to provide a secure electricity trading system that is decentralized and preserves privacy for EVs during the electricity trade.

#### 3.2.1. Blockchain Architecture

**Figure 5:**
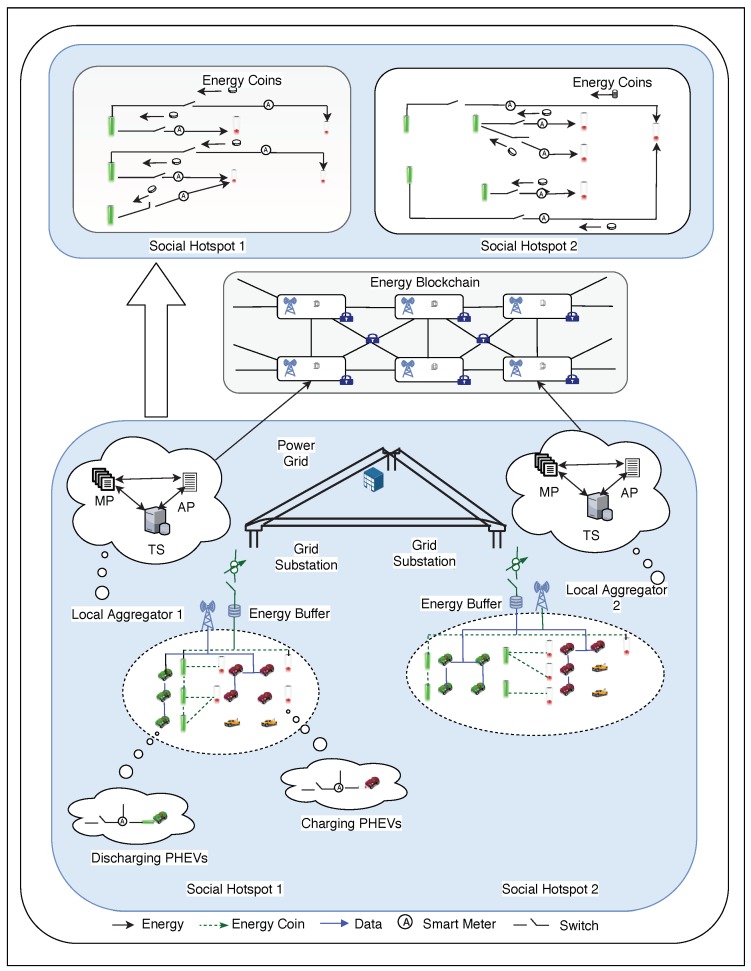
Architecture for energy trading in electric vehicles.

A blockchain-based solution to trade electricity brings with it the advantages of security, decentralization, and trust. A system known as PETCON is designed by the authors of [52] to achieve secure trading of electricity. Among the other existing works on energy trade in EVs, this architecture is not only shown to be secure against cybersecurity attacks but also shown to be cost-optimized and scalable for multiple nodes, as shown in Table 3. Further, it is based on the P2P architecture discussed in the previous section on the P2P energy trade. Consortium blockchain technology is used here because of the cost advantage compared to the existing blockchain methods employed in electricity trading. Based on this model, a generic architecture for electricity trading among EVs is shown in Figure 5.

The EVs in this system are divided into three categories as follows:

- Discharging EVs, which sell surplus energy.
- Charging EVs, which demand energy.
- Idle EVs, which neither demand nor sell energy.

An EV joins the system after registering itself with the trusted authority and chooses its role as a discharging/charging EV as per its current energy states and the future energy requirements. The charging EV sends a request to the EAG, which broadcasts its demand to the discharging EVs. Upon receiving their responses, the EAG performs bidding and transactions among the EVs. The payment of energy coins is made by the charging EV to the discharging EV’s wallet address, which verifies it using the last block in the memory pool of the EAG. The fastest EAG is considered as the leader of the consensus process and sends data and timestamp of the block along with the PoW to other EAGs for verification and auditing. Only when all the EAGs reach an agreement are the data stored as a block in the blockchain. Since a consortium blockchain is being used here, the number of nodes in the network is not expected to grow a lot unlike in a public blockchain. For networks with the exponential growth of nodes, running PoW will require high energy consumption. Instead, other consensus algorithms such as Proof-of-Stake (PoS) [56], Proof-of-Burn (PoB) [57] and Proof-of-Elapsed-Time (PoET) [58] may be run.

The various entities used in the energy blockchain are discussed here. Borrowers are the energy buyers borrowing energy coins from the credit banks. Transaction Servers (TS) are responsible for collecting and counting the energy requests and matching the transaction pairs for energy trading. Wallets are the entities that store the energy coins. Account pools (AP) are the entities that record the wallets, the energy coin accounts, and the wallet addresses. The transaction records of the local EVs are stored in the Memory Pools (MP). Credit banks are the entities through which borrowers borrow energy coins based on their credit values.

#### 3.2.2. Block Structure

**Figure 6:**
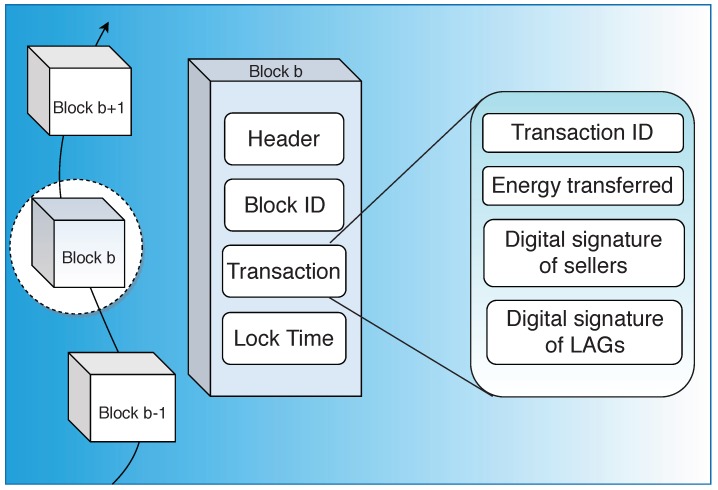
Block structure of energy trading in electric vehicles.

Information about the traded electricity and the transaction records of the digital assets are stored in a block of this blockchain. The transaction records are collected and managed by the local energy aggregators (EAGs). These records are encrypted, signed with digital signatures and audited by the rest of the EAGs using the consensus algorithm, PoW. The block structure is shown in Figure 6. The first is PoW for EAGs, in which data auditing by authorized EAGs is done. Various EAGs compete for the creation of blocks by finding PoW and the fastest one audits the transaction records and puts in the block which is verified by other EAGs. Secondly, PoW for EVs is the amount of energy sold by that EV, which is measured and recorded by the built-in smart meters.

#### 3.2.3. Technologies Used

- Smart contracts: These are used for the commitment from the charging EVs before the local aggregator (LAG) makes the contract with the discharging EVs. A deal is formed, which includes the rates for the purchase of battery and discounts for charging and parking. Breaking the terms of contracts will lead to penalties. Smart contracts are used for registering the EVs and for securing the energy trade (e.g., detection of malpractices).
- Digital currency: The NRGcoin is the digital currency used in energy trading between the EVs. After the trading of electricity, there will be a transfer of NRGcoins from the wallet of charging EV to the discharging EV’s wallet address.
- Double auction mechanism: Double auction mechanism is used for energy negotiation, bidding, and transactions between the EVs. This mechanism is used for price optimization and also for optimizing the electricity units traded between EVs, thus maximizing social welfare along with the privacy protection of EVs. LAG acts as an auctioneer and performs this mechanism iteratively according to the selling prices of discharging EVs and buying prices of charging EVs. The auctioneer will determine the final prices of the trading and the amount of electricity to be traded, which will be useful to protect the EV information during the electricity trade [51].

### 3.3. Security and Privacy-Preserving Techniques

Smart meters in the smart grid are placed at every house to get information about electricity consumption in real-time, which is used by the utilities for various purposes [59,60]. By analyzing the electricity consumption profile of the users, malicious entities can track the electricity usage pattern, thereby disclosing the users’ private information [61,62,63]. The authors of [64] proposed a blockchain-based scheme for efficient data aggregation and privacy preservation. In their work, they divided the users into many groups with each group using a blockchain for recording the users’ data. Bloom filter is used by the scheme for fast authentication, to facilitate a quick check of the legality of the user ID in the system. For the preservation of privacy within the group, pseudonyms are used by the users. Although blockchain technology does not directly ensure privacy preservation, advanced cryptographic mechanisms can be incorporated for enabling data privacy. Zero knowledge proof (ZKP), Elliptic Curve Digital Signature Algorithm (ECDSA), and linkable ring signatures are some of the techniques which can protect the privacy of the devices involved. The privacy-preserving blockchain architecture discussed here makes use of ZKP combined with pseudonyms for the users.

#### 3.3.1. Blockchain Architecture

**Figure 7:**
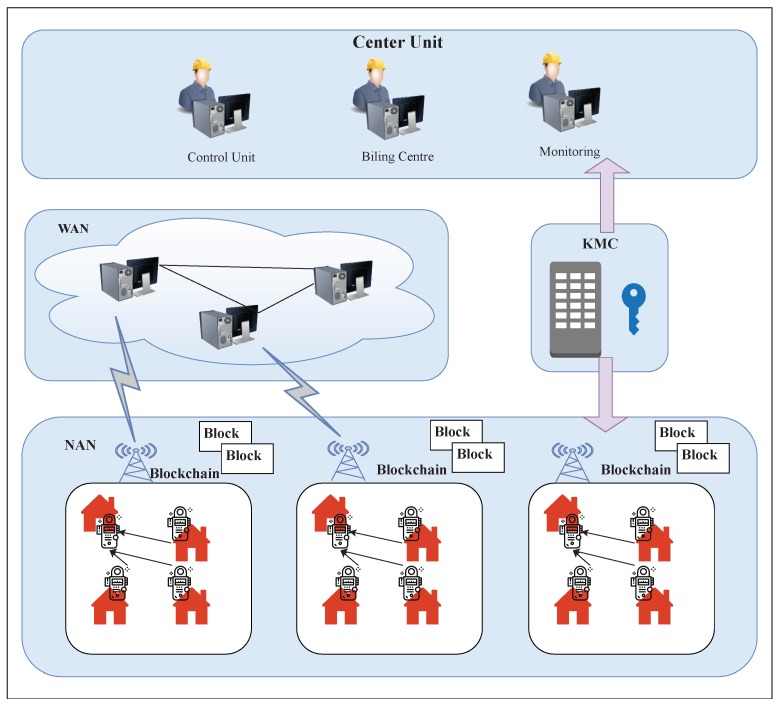
Architecture for data aggregation and privacy preservation scheme.

A typical blockchain-based architecture for the data aggregation scheme is described in Figure 7. This is based on the reference model presented by Guan et al. [64]. We present a comparison of blockchain-based state-of-the-art research works done on security and privacy-preserving techniques in the smart grid in Table 4. Based on electricity consumption, users are divided into various groups/neighborhood area networks (NANs). Multiple public and private key pairs are generated by the key management center (KMC) for every user using RSA, a popular public-key cryptography algorithm, with the pseudonym of the user being the public key. By collecting the pseudonyms, the bloom filter is created by the KMC for every group and sent to all the users of the corresponding group. The authenticity of the user pseudonym can be verified using zero-knowledge proof, a probabilistic verification method in cryptography. At every time slot, a mining node is selected among the users of the group based on the average consumption of the electricity data. The mining node aggregates the electricity data consumed, records them in a private blockchain and sends it to the central unit with the help of wide-area network (WAN). The central unit can extract the electricity consumption profile in real-time for energy planning and dynamic pricing. The billing center on the arrival of the billing date calculates the users’ electricity bill and records it in the blockchain.

#### 3.3.2. Block Structure

**Figure 8:**
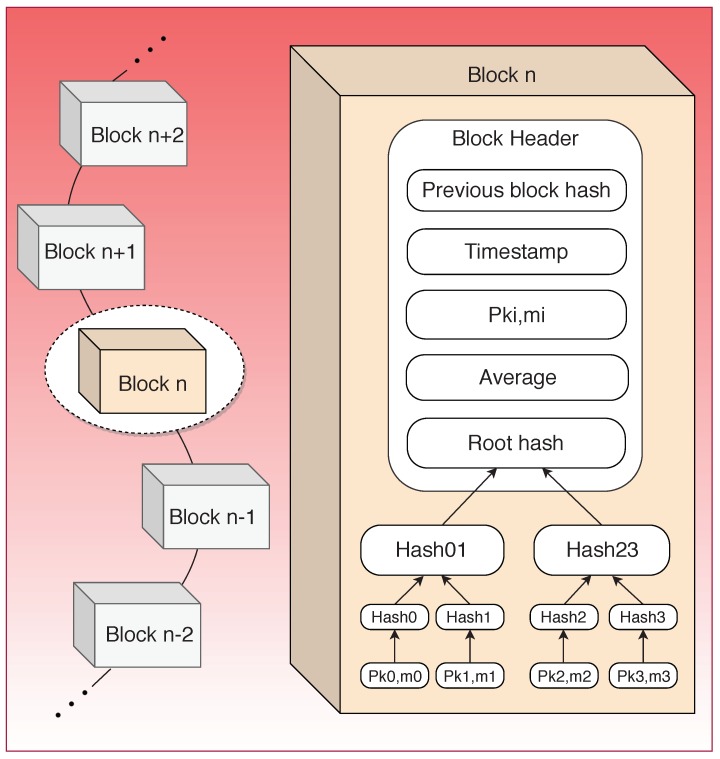
Block structure for data aggregation and privacy preservation scheme.

The block structure for this scheme is shown in Figure 8. The PoW consensus algorithm is adopted for the selection of mining nodes among the different users [64]. The mining nodes record the electricity consumption data in a Merkle tree. Block header records the hash value of the previous block, the root hash value of the Merkle tree, timestamp, pseudonym and the average. Timestamp marks the time at which each transaction occurs on the blockchain and indicates when and what happened in the blockchain. A pseudonym is a public key for that user, generated by the KMC. Average provides the value of the average consumption of electricity data.

#### 3.3.3. Technologies Used

The technologies used in this scheme can be discussed in two aspects, namely the preservation of user identity and preservation of user data. User identity can be preserved using a virtual ring, where the control center validates the user’s identity using the ring signature without actually knowing its identity. In smart contracts, the system can be protected against any theft by using multiple independent parties to sign the transactions before considering them as valid (multi-signature transactions). User identity can also be preserved using pseudonyms or based on household battery. Depending on the consumption profile of the household, the battery will discharge (if household consumption goes high). Hence, using the household battery, the electricity consumption profile of the user can be balanced and at the same time, the privacy of the user can be protected. The other technique to preserve the user data is based on authentication in which the credentials generated by the consumer are sent for his/her proof of identity to the control center, which signs the credentials. User data can also be preserved based on data aggregation, which employs data obfuscation and homomorphic encryption techniques. In data obfuscation, noise is added to the original data to make data of the user’s electricity consumption unclear, while, in homomorphic encryption, the intermediary agent is allowed to operate on encrypted data without any information of the plaintext.

### 3.4. Power Generation and Distribution

Numerous cyberattacks on smart grids have been undertaken in the past where the malicious attackers have used various methods such as Denial of Service (DoS), Data Injection Attacks (DIA), etc. to manipulate data and gain control in the grid [68,69]. This has resulted in complications such as regional power outages and even complete blackouts [70]. Incorporating blockchain into the power generation and distribution systems help in the prevention of data manipulation since one of the prime characteristics offered by the blockchain system is its ability to ensure data immutability.

#### 3.4.1. Blockchain Architecture

**Figure 9:**
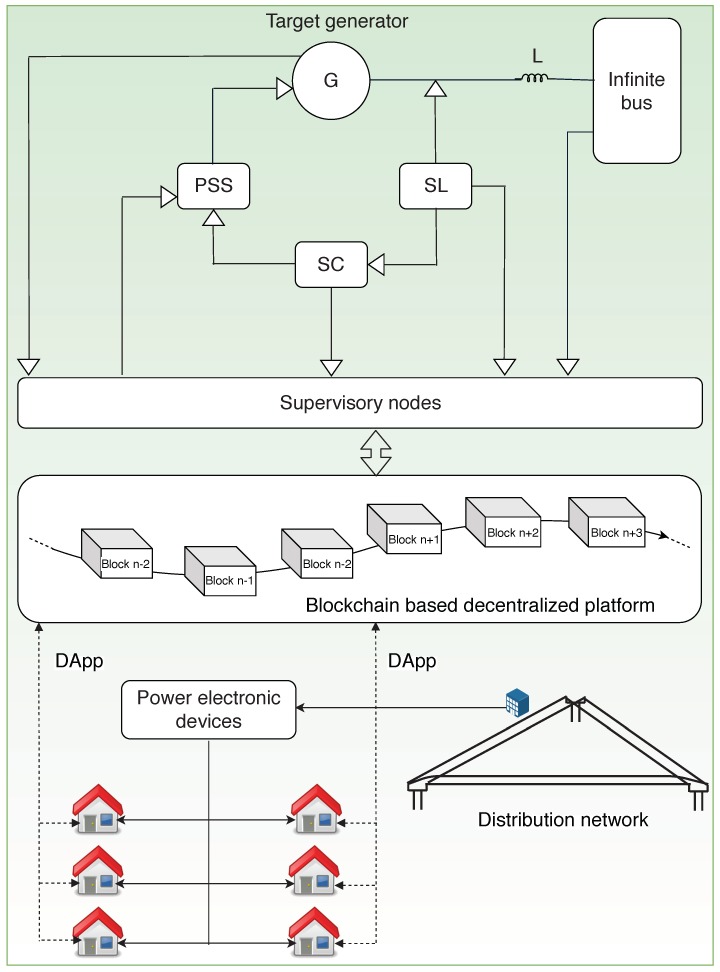
Architecture for power generation and distribution.

Figure 9 shows how a blockchain system can be incorporated into a power generation station with a Single Machine Infinite Bus (SMIB) system and its distribution networks. This framework is created based on the architectures discussed in other works on power generation and distribution using blockchain [66,71].

An SMIB is constituted by a synchronous generator G, which is connected to the infinite bus through a reactance Z; a load, which is fed through a load switch SL; a Power System Stabilizer (PSS) used for damping the generator’s electro-mechanical oscillations to protect the shaft line and to provide grid stabilization; and a control switch SC for the PSS, which takes its input from the load switch. A cyber attacker can use a suitable attacking scheme to modify the conditions of the switches resulting in the removal of load from the generator and leading to sudden transition in the terminal voltages to very high values. Since the control switch, SC to PSS, is tampered with, automatic voltage regulation is rendered unresponsive and damping of the oscillations does not occur. This leads to shaft damage and loss of synchronization in the target generator. This can be avoided by incorporating the blockchain into the power generation system [71]. The time-stamped values of each switch state and the target generator can be stored as data in the blocks. Specific nodes can be given the privilege to validate and mine the data into the blocks. In the event of an attack, violation in the current state of each switch should be reported to the blockchain. A smart contract in the metering device would then identify the violation and maintain the previous terminal value of the target generator by enforcing PSS to damp the oscillations.

#### 3.4.2. Block Structure

**Figure 10:**
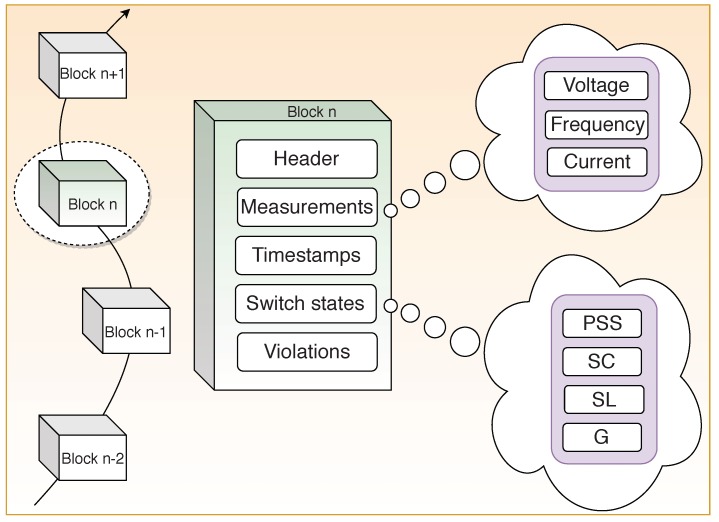
Block structure for power generation and distribution.

The block body, as shown in Figure 10, includes measurements, switch states, violations and timestamp. The measurements part of the block includes the frequency, voltage, and current generated by the system. Switch States store the states of the switches SL, SC, and PSS and the measured value of the target generator G. The failed status of the switches as reported by the respective metering devices is stored in the violations part of the block. The timestamp indicates the time instant of the measurement. This data is further utilized by the smart contract to take the necessary action.

#### 3.4.3. Technologies Used

Decentralized Applications (dApps) are the applications running on a network of peers. They can be utilized in blockchain-based smart grid systems to connect every prosumer, consumer and energy substation in the grid [72]. Smart contracts are used to intelligently make decisions and to monitor data. In a DoS attack on any node of the distribution system, a massive amount of data is sent from multiple sources rendering the node unresponsive. If it occurs on a Remote Terminal Unit (RTU), which is responsible for monitoring and control of the SCADA devices in the grid, the whole balance in the distribution system is disrupted. Since the RTU will not be able to communicate with the master controller in such circumstances, there may be an excess supply of power leading to blowing up of fuses or to variations in frequency. The dApps provide a means of remotely monitoring the power consumption metrics, to monitor the change in values of voltage and frequency measures by their direct links to the blocks in the blockchain network. Since the measured metrics such as the voltage, current, and frequency are stored in the blocks with timestamps, these values can be compared against the measurements in the grid to identify the attack and verify the real power consumption.

### 3.5. Secure Equipment Maintenance for Smart Grids

Equipment health monitoring, fault diagnosis, and maintenance are integral parts of the smart grid system. Traditional methods of diagnosis involve the necessity for technicians to visit the field for diagnosis and maintenance. It also involves an investment of manpower and other expenses at the risk of the client being unsatisfied with the services. This leads to a need for developing systems that can reduce the maintenance time and are unaffected by regional restrictions. Smart grids encompass several types of equipment ranging from substations to smart meters installed in homes. Such a complex smart system, in turn, requires smart equipment maintenance measures to ensure high efficiency and reliability [73,74].

#### 3.5.1. Blockchain Architecture

**Figure 11:**
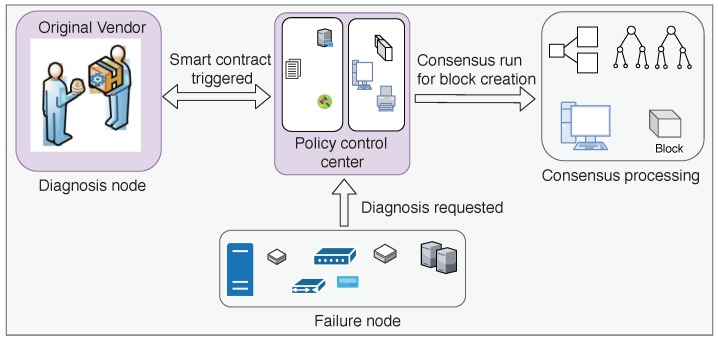
Architecture for secure equipment maintenance.

A blockchain integrated framework, as shown in Figure 11, can be utilized to create a platform for interaction among the vendors, diagnosis depots and clients in a secure manner to decide upon the required maintenance measures for a mutually agreeable price. This framework has been chosen from the existing works in smart grid equipment maintenance and monitoring using blockchain technology, as discussed in Table 5. Zhang and Fan [75] used a consortium blockchain with pre-determined book-keeping nodes to implement this system.

Whenever a piece of electrical equipment exhibits some fault or abnormality in its operation, a request for diagnostic services is sent in the network. These equipment are then labeled as failure nodes in the network. Smart contracts are modeled to respond to the failure nodes and to lead the flow of operation as decided. The failure node needs to deposit virtual currency for the maintenance services to the smart contract. If the equipment is within its warranty period, its vendor will perform the required services and the deposited currency will be returned to the node. However, if the device is out of its warranty period, the vendor and the other verified maintenance teams who are willing to tend to the diagnosed fault will now bid to obtain the tender. These nodes will form the diagnosis nodes. Only the entities that are validated and registered in the system can compete as diagnosis nodes. They compete fairly in the auction process and the smart contract decides upon who gets the bid depending on the auction algorithm. When the deal is finalized, the smart contract broadcasts the transaction with the details fed in the block format to the network. The bookkeeping nodes carry out the respective consensus algorithm to add the block to the network. This prevents double-spending and once a consensus is reached regarding the bid, neither of the parties can withdraw without depositing penalty, making it a reliable system.

#### 3.5.2. Block Structure

**Figure 12:**
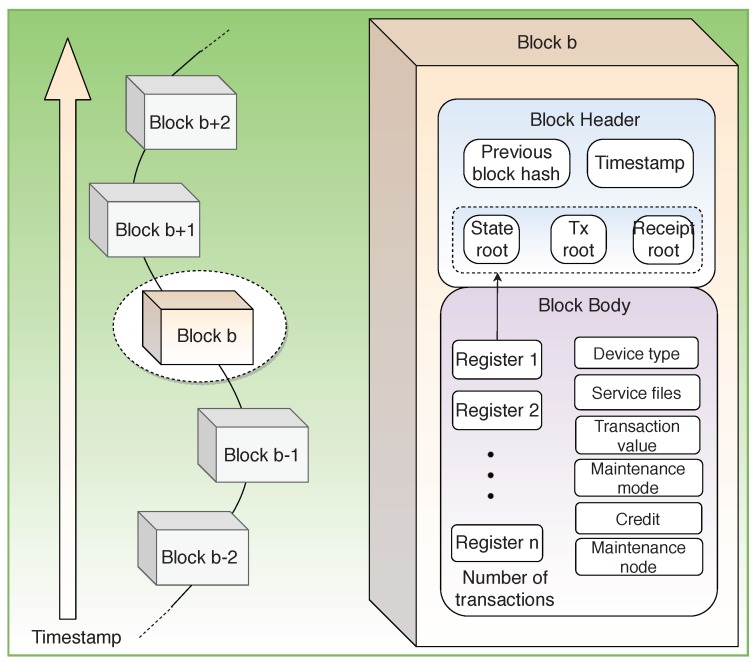
Block structure for secure equipment maintenance.

As shown in Figure 12, the block body consists of Device Type; Transaction Value denoting the cost of the maintenance; Diagnosis Node, which responds to the request for diagnosis; Service Files with information related to the failure node, failure type, time, etc.; Maintenance Mode, indicating whether it is a remote or on-site maintenance; and Credit, which acts as a means for one node to assess if they require the services of another.

#### 3.5.3. Technologies Used

To provide satisfactory customer service, through a smartphone application that communicates with the blockchain network via the smart contract, one can connect and log into the network. The app can receive the progress of the transaction regularly and can be used as a tool to bring about any adjustments in the policy of operation as required. Information about the node initiating the diagnosis, the diagnosis method adopted, the payment information, etc. can be relayed periodically to the app, which leads to an even more reliable and secure transaction.

The comparison between the different use cases discussed above is described in Table 6.

## 4. Commercial Implementations of Blockchain in the Smart Grid

One of the foremost applications of blockchain in the smart grid is to incorporate virtual currencies for payments. The first company to accept Bitcoin for payment of energy bills was BASNederland [76]. This inspired several other companies to come up with cryptocurrency-based solutions for billing and metering, and several of them providing incentives for users making payments using cryptocurrency instead of those using fiat currencies [77,78]. Meanwhile, some other companies such as the South Africa based startup Bankymoon are developing smart meters with integrated payments using Bitcoin [79]. The Netherlands based companies Spectral and Alliander have developed a blockchain-based token for energy sharing called Jouliette [80]. This token allows the P2P transaction of electricity through spending the energy tokens from their e-wallets. Another company, PowerLedger, an Australia based startup, developed a blockchain-based platform for P2P renewable energy transfer between residential prosumers and consumers [81]. The platform makes use of a smart contract-based system called POWR to enable the transfer of tokens called Sparkz. The company has demonstrated its ability in saving significant revenue for the users and supplying additional incentives for renewable energy producers.

The most significant implementation of blockchain in P2P decentralized energy trading and creation of a local marketplace is the Brooklyn microgrid. It was launched by the US energy firm LO3Energy along with ConsenSys, a Blockchain company [82]. The first trial of the project, which was carried out with five prosumers and five consumers, marked the first-ever recording of energy transactions using blockchain. Ethereum-based smart contracts were used to architect the platform, which facilitated the consumers to buy surplus renewable energy from the prosumers through a token-based transaction system. The surplus energy tapped through the rooftop photovoltaic (PV) panels by the prosumers is converted into tokens by the smart meters installed in their houses, which can be directly used for trade in the energy market. This platform records the mode of transaction in energy units or tokens as per the requirement of the user. The ledger stores, in chronological order, details about each transaction, such as the parties involved, the amount of energy consumed/sold and the related contract terms. The future developments in the Brooklyn microgrid system include assigning the users with the ability to choose from the prospective buyers/sellers the required energy, among other privileges such as the ability to decide the percentage of energy share needed to buy from prosumers and the main grid. A bidding system will be used in which renewable energy will be sold to the highest bidder. A mobile application is also being developed to provide users with easy means of interaction with the platform. Such projects will change the face of energy transactions in the coming future [83].

ShareandCharge is a blockchain-based platform developed jointly by InnogyMotionwerk, a subsidiary of German energy conglomerate RWE, and a blockchain firm Slock. This platform allows P2P energy trading among EVs and the private charging stations [84]. The users can use their e-wallets to know about the real-time prices and carry out transactions on this public Ethereum-based platform, which automatically manages certificates and billing. JuiceNet is yet another blockchain-based platform deployed by a company called eMotorwerks in California for leasing out charging piles to EV drivers for some time [85]. The platform maintains a record of the transactions and allows the owner of the charging pod the required payment. Moreover, JuiceNet provides a mobile application for the owners of the EVs to locate a charging pile from among the enlisted charging piles in the neighborhood.

## 5. Challenges for Blockchain Incorporation into Smart Grid

### 5.1. Scalability Issues

Transactions in a blockchain increase on a day-to-day basis, which calls for heavy storage capabilities to accommodate the ever-growing number of transactions. Currently, the storage for Bitcoin has exceeded 200 GB while that for Ethereum has reached about 1 TB. Even though a considerably high number of transactions are being carried out using Bitcoin, the processing rate of data into blocks in a blockchain is estimated to be about seven per second. Meanwhile, the average number of transactions in Ethereum is up to 15 per second. Such low rates of processing are attributed mostly to the consensus mechanism, PoW, which is used in the Bitcoin technology. High processing power and time are required by the nodes to compute the PoW algorithm to add the block into the blockchain network. According to the report in [86], to process 30 million transactions, 30 billion kWh of electricity was spent, which accounted for about 0.13 percent of global electricity consumption. In the energy sector, for large scale operations, the number of transactions per second is very high since thousands of users are simultaneously involved in the process of buying and selling energy. This creates a large overhead upon the nodes involved in the consensus and validation process. This problem can be addressed by replacing the PoW consensus algorithm either with the Proof-of-Stake (PoS) or the Proof-of-Authority (PoA) algorithm. These algorithms require much less computing capacity and support much higher rates of transactions. A new blockchain platform named EnergyWeb blockchain is aimed specifically at the energy sector with transaction rates as high as a few thousand per second. It uses the PoA consensus mechanism, which gives it such high processing rates. Further research and innovations have to be carried out to find solutions to properly scale up the platform to accommodate the requirements of the smart grid system without compromising on the security aspects [13]. Other so-called “second-layer” solutions are intensely being researched by the community for addressing the scalability issues [32,87]. Off-chain [88] and side-chain [89] techniques have been proposed for reducing the number of transactions and for parallelizing the transaction validation, respectively. Research is also leading to advancement in the enabling technologies such as Distributed Hash Table (DHT) [62], InterPlanetary File System (IPFS) [90], and nonlinear block organizations such as Directed Acyclic Graph-based chains (DAGchains) [91] to potentially address the scalability and throughput challenges.

### 5.2. Chances of Centralization

Currently, blockchain application in the energy sector is still a budding technology and is prone to attacks from the energy conglomerates who might exploit it for financial advantages. One of the reasons for centralization is the clustering of mining nodes into mining pools for better computational capacity. The only chance of changing the transactional data in a block is through the 51 % attack, where the attacker controls 51 % of the computational capacity in the network. By clustering the mining nodes into pools, there exists a risk of the mining pools acquiring enough resources to plot a malicious attack. Another reason for centralization is the fact that much of the architecture in the energy sector is based on consortium or private blockchains. The reason for their popularity is the problem of power wastage and latency associated with public blockchain architectures. Since a predefined set of nodes are responsible for validation and consensus in the public blockchains, chances of malpractice exist. Therefore, strict supervision under governmental laws should be enforced especially in the beginning stages to ensure security.

### 5.3. Development and Infrastructure Costs

Implementing blockchain in the smart grid requires high infrastructural costs for re-architecting the current grid networks, upgrading smart meters to aid in transactions through smart contracts, infrastructure for Information and Communication Technologies (ICT) specific for Blockchain operations, other related Advanced Metering Interfaces (AMI) and software for development of the whole platform. Such high infrastructure costs may dissuade grid operators from the incorporation of blockchain into the grid structure. The current infrastructure of the grid has been adopted after years of research and development and it yields optimal results with much less overall expenditure. For example, the grid communication system currently employs technologies such as telemetry which is more mature as well as much less expensive compared to blockchain.

### 5.4. Legal and Regulatory Support

The regulatory bodies do support the active participation of users in the energy market, and the formation of community energy structures. However, when it comes to radical changes in the main power grid framework, the current grid legal system does not support the trading of energy from prosumers to consumers and does not endorse the adoption of the distributed ledger into the framework. New types of contracts have to be developed especially for the P2P trading system and changes in the energy tariffs need to be brought about to support such services. Such matters are heavily regulated in the current grid system. For these reasons, even though blockchain technology has proven its worth in the formation of microgrids, without amended legal structures, it is very challenging to adopt the technology into the main grid framework.

## 6. Future Research Directions

- Using blockchain technology, a decentralized computing platform can be created in addition to the trading infrastructure. All the peers who participate in the network give a share of their computational capacity, thus increasing the total capacity within the system, which will enable efficient operation and control of microgrids. It will also increase trust among the owners and the scalability of the grid. Even if an extra consumer is added, there will be no increase in complexity.
- Any new technology needs to prove that it can offer the scalability, speed, and security required before it can be widely accepted. The blockchain technology has already passed the proof of concept but it still needs to be scaled up and be cost-efficient. For grid communication, there already exists established solutions such as telemetry, which are significantly cost-efficient. Blockchain technology also has to compete on all the above-mentioned aspects for wider utility and acceptance. There is a scope in cutting the cost for data storage by storing actual data in the sidechains (as subsidiary blockchains) and operating the main blockchain as a control layer rather than as a storage layer.
- In current large-scale blockchain networks such as Ethereum or Bitcoin, any upgrade to the software code that runs in the participating nodes must be approved (via consensus algorithms) to be affected throughout the network. Any disagreements to do so can lead to forking and fragmentation of the network, compromising its security and data integrity. The design of these blockchain networks for smart grids must be protected against such effects.
- Blockchain-based solutions for various aspects of decentralized grid management and control, e.g., improving demand–supply balance, automated verification of grid assets, forecasting grid requirements, self-adjusting power consumption based on price surges or drops, etc., need to be explored.

## 7. Conclusions

The smart grid is a booming technology in the energy sector and it essentially needs a reliable and secure framework for operations. This paper discusses several use cases in which blockchain can be incorporated into the smart grid to open the doors to a wide range of possibilities. P2P energy trade is the need of the hour for promoting sustainable use of energy and for tapping renewable energy sources. With the advent of using the blockchain in the smart grid scenario, V2V and V2G energy transfer have become simpler and more reliable than ever. Although the privacy requirement is not directly ensured by blockchain technologies, advanced cryptographic techniques such as ZKP and ECDSA can be incorporated to enable privacy preservation. Further, this paper discusses the data immutability aspect of blockchain, which can be used to prevent cyber attacks, especially in the power generation and distribution systems. A secure equipment maintenance system that ensures efficient diagnosis using smart contracts is also discussed. Along with discussing various application areas of blockchain, this paper illustrates potential blockchain architectures and block structures for each of these areas. However, for the widescale adoption of blockchain in the smart grid, the industry and research community will have to work together to address the significant challenges that lay ahead.

## Figures and Tables

**Table 1:** Classification of blockchains [25,26,27,28,29,30,31].

Parameter	Public Blockchain	Consortium Blockchain	Private Blockchain
**Receptivity**	Fully open	Open to some nodes	Open to a person/entity
**Access to Write**	Anyone	Specific nodes	Internally controlled
**Access to Read**	Anyone	Anyone	Open to the public
**Obscurity**	More	Less	Less
**Speed of Transaction**	Low	High	Extremely high
**Decentralization**	Fully decentralized	Less decentralized	Less decentralized

**Table 2:** Comparison of state-of-the-art research papers on P2P energy trading using blockchain.

Ref.	Cost and Energy Optimized	Optimization Applied	Secure against Attacks	Security Analysis	Scalable	Performance Analysis
[41]	✓	✓	✗	✗	✗	✗
[42]	✓	✓	✗	✗	✓	✗
[43]	✗	✗	✓	✗	✓	✓
[44]	✓	✓	✗	✗	✓	✓
[38]	✓	✓	✓	✓	✓	✓

**Table 3:** Comparison of state-of-the-art research papers on energy trading in Electric vehicles (EVs) using blockchain.

Ref.	Cost and Energy Optimized	Optimization Applied	Secure against Attacks	Security Analysis	Scalable	Performance Analysis
[53]	✗	✗	✓	✗	✓	✓
[54]	✓	✓	✗	✗	✗	✗
[55]	✓	✓	✓	✓	✓	✗
[52]	✓	✓	✓	✓	✓	✓

**Table 4:** Comparison of state-of-the-art research papers on security and privacy-preserving techniques in smart grid using blockchain.

Ref.	Cost and Energy Optimized	Optimization Applied	Secure against Attacks	Security Analysis	Scalable	Performance Analysis
[65]	✓	✗	✗	✗	✗	✗
[66]	✓	✓	✓	✓	✗	✗
[67]	✓	✗	✓	✓	✓	✓
[64]	✓	✓	✓	✓	✓	✓

**Table 5:** Comparison of state-of-the-art research papers on equipment maintenance and monitoring in smart grids using blockchain.

Ref.	Cost and Energy Optimized	Optimization Applied	Secure against Attacks	Security Analysis	Scalable	Performance Analysis
[45]	✗	✗	✓	✗	✗	✗
[66]	✓	✓	✓	✓	✗	✗
[67]	✓	✗	✓	✓	✓	✓
[75]	✓	✓	✓	✓	✓	✓

**Table 6:** Summary of blockchain applications in the smart grid.

Application	Problem Addressed	Preferred Blockchain Architecture	Sample Block Content	Technologies Used
P2P energy trading	Decentralized electricity trade between prosumers and consumers, promotion of renewable energy harvesting	Consortium blockchain	Transaction ID, consumer meter ID, amount of energy requested and energy granted, a digital signature of the seller and the processing node	Smart contracts, virtual currency, credit-based e-wallet
Energy trade between EVs	Buying and selling of surplus energy between EVs, privacy-preserving of EVs	Consortium blockchain	Transaction ID, EV’s meter ID, charged energy, a digital signature of the charging station and the processing node	Smart contracts, energy coins
Security and privacy- preserving techniques	To protect the application usage pattern and the privacy information of users	Private blockchain	Transaction ID, the energy transferred, a digital signature of the seller and the LAGs	Bloom Filter, data aggregation, authentication techniques
Power generation and distribution	Protection from cyber attacks, incorporation of abnormality control measures	Consortium blockchain	Time of measurement, measurement of frequency, voltage and current, switch states	Smart contract, dApps, remote control of distortion using power electronics devices
Secure equipment maintenance	Platform for interaction between vendor and client for equipment diagnosis and privacy preservation	Consortium blockchain	Device ID, mode of maintenance, service files and credits, transaction value	Smart contracts, user interaction using smart phone app
